# Sandwich strategy for the treatment of kyphosis with scoliosis and bilateral hip ankylosis in ankylosing spondylitis: a case report

**DOI:** 10.3389/fsurg.2026.1802819

**Published:** 2026-05-29

**Authors:** Shihao Hong, Zihe Niu, Baipan Zhang, Pengyu Bao, Yu Wang, Xin Yang

**Affiliations:** Department of Orthopedics, Peking University First Hospital, Beijing, China

**Keywords:** ankylosing spondylitis, arthroplasty, kyphosis, pedicle subtraction osteotomy, scoliosis

## Abstract

Ankylosing spondylitis can present with severe kyphosis, scoliosis, and bilateral hip ankylosis, posing significant therapeutic challenges. Conventional “hip-first” or “spine-first” strategies are often limited by interdependent biomechanical constraints. This case report describes a successful staged “sandwich” protocol in a 48-year-old male with ankylosing spondylitis, thoracolumbar kyphosis, scoliosis, and bilateral hip ankylosis. The treatment involved three sequential stages: first, right total hip arthroplasty; second, pedicle subtraction osteotomy from T10 to L4; and third, contralateral total hip arthroplasty. This approach addressed critical issues including intraoperative positioning, global spinopelvic realignment, and individualized acetabular component orientation. Postoperative radiographs demonstrated marked improvement in spinal sagittal and coronal alignment, with well-positioned hip prostheses. The “sandwich” strategy adheres to a coherent biomechanical principle of “unlock–rebalance–match,” systematically restoring mobility, correcting alignment, and ensuring long-term prosthetic stability. This approach offers a promising alternative for managing complex hip-spine syndrome in advanced ankylosing spondylitis.

## Background

Ankylosing spondylitis (AS) is a chronic immune-mediated inflammatory disorder predominantly affecting the spine and sacroiliac joints. It is clinically characterized by symptoms such as chronic low back pain, morning stiffness, and progressive spinal immobility (1). The underlying pathological hallmarks include persistent inflammation, structural bone destruction, and aberrant new bone formation leading to syndesmophyte development and eventual ankylosis (2). As the disease progresses, some patients develop severe thoracolumbar kyphotic deformities and bilateral hip involvement, often progressing to complete ankylosis of the hip joints (3). This results in marked limitation of joint mobility, significant impairment of posture and gait, reduced quality of life, and, in severe cases, substantial psychological distress due to functional disability and physical disfigurement.

Pedicle subtraction osteotomy（PSO） has become a well-established surgical approach for correcting rigid spinal deformities in AS, capable of restoring sagittal alignment and improving pulmonary function and forward gaze (4). When combined with total hip arthroplasty (THA), it can effectively address concomitant hip pathology, alleviate pain, restore joint function, and enhance overall mobility and activities of daily living (5).

However, optimal surgical management remains controversial in complex cases involving concomitant severe kyphotic spinal deformity and bilateral hip flexion contractures—particularly regarding the sequence of interventions: whether to prioritize hip reconstruction (“hip-first”) or spinal correction (“spine-first”). Each strategy presents distinct biomechanical and clinical limitations, and currently, there is a paucity of evidence or consensus on surgical planning for patients with additional complicating factors such as scoliotic spinal deformities (6, 7).

To address this complex clinical scenario, we report a case managed successfully using a staged “sandwich” protocol: unilateral THA followed by PSO, and finally contralateral THA. This sequential approach allowed safe positioning for spinal surgery while preserving long-term hip stability and optimizing global sagittal balance. In this report, we describe the surgical rationale, biomechanical considerations, and clinical outcomes of this strategy, aiming to provide insight into its potential role in the management of highly challenging, multi-compartmental deformities in advanced ankylosing spondylitis.

The novelty of this report lies in applying this staged logic to a patient with simultaneous thoracolumbar kyphosis, coronal scoliosis, and bilateral bony hip ankylosis. In contrast to previously reported “spine-first” or “hip-first” algorithms, the first arthroplasty was used selectively to create a safer operative position rather than to provide final spinopelvic normalization, whereas the last arthroplasty was intentionally postponed until pelvic orientation had stabilized after spinal correction (8, 9).

## Case presentation

A 48-year-old male presented with a 30-year history of bilateral hip pain and progressive functional impairment, diagnosed with AS at age 18. His Ankylosing Spondylitis Quality of Life (ASQoL) score was 16/18, indicating profoundly impaired health-related quality of life. Physical examination revealed no joint swelling; however, both hips were fixed in flexion contracture with complete loss of active and passive range of motion (ROM). The left femur exhibited mild external rotation, whereas the right demonstrated mild internal rotation. Harris Hip Score (HHS) was 48 on the left and 49 on the right—both classified as “poor.” Visual Analog Scale (VAS) pain scores for both hips were 3/10 at rest. The physiological spinal curvatures were absent, with evident thoracolumbar kyphosis and C-shaped scoliosis. Spinal mobility was severely restricted in all planes. No significant tenderness was noted over the spinous processes or paraspinal regions.

**Figure 1:**
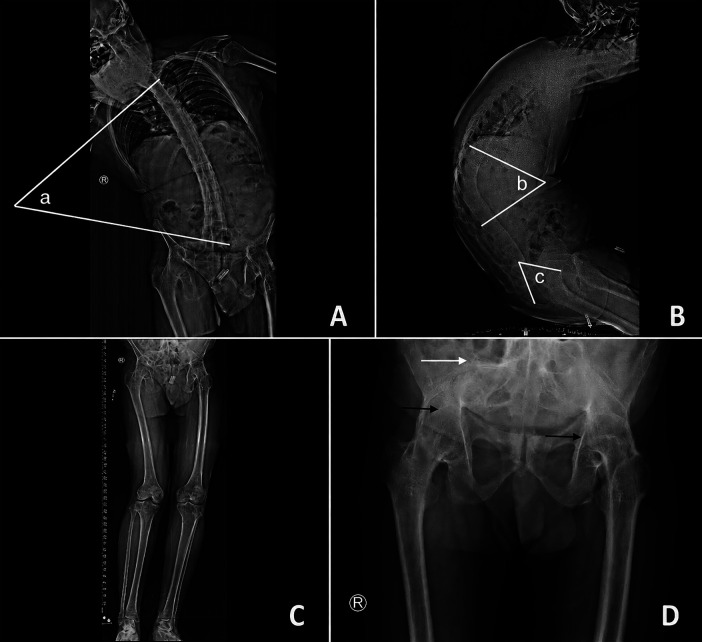
Preoperative x-ray films showed **(A)** bamboo-like changes in the vertebral bodies in the coronal plane of the spine, with the spine bending to the right, cobb angle of a = 50°; **(B)** kyphosis in the sagittal plane of the spine, cobb angle of b = 55°, PI of c = 55°; **(C)** full-length standing lower limb radiographs showing bilateral hip ankylosis, with both hip and knee joints in flexion; **(D)** bilateral hip orthopantomogram showing disappearance of the sacroiliac joint space and bony fusion (white arrow), both hip joints demonstrate total bony ankylosis (black arrows).

Radiographic evaluation revealed the following findings:

1. Lateral whole-spine radiograph demonstrates thoracolumbar kyphosis with a Cobb angle of approximately 55°. Vertebral bodies exhibit characteristic “bamboo spine” morphology. The pelvic incidence (PI) measures 55°. Anteroposterior (AP) whole-spine radiograph reveals a structural C-shaped scoliosis with a Cobb angle of 50° at the thoracolumbar junction.
2. Standing long-leg AP radiograph shows bilateral ankylosis of the hip joints, accompanied by flexion contractures at both hips and knees. Additionally, there is external rotation of the left femur and internal rotation of the right femur.
3. AP pelvic radiograph confirms bilateral sacroiliac joint fusion, manifested by complete obliteration of the sacroiliac joint spaces (indicated by white arrow). Concurrently, both hip joints demonstrate total bony ankylosis (marked by black arrows) (Figure 1).

Diagnoses:

1. AS accompanied by kyphosis
2. Scoliosis
3. AS with bilateral hip involvement

Surgical Procedure:

Stage 1. Right THA: The patient was placed in the left lateral decubitus position, and a lateral incision was made on the right hip joint. Layer-by-layer dissection was performed to expose the joint capsule and hip joint. Osteotomy was performed 1 cm above the lesser trochanter, and the soft tissues around the acetabulum were released. The acetabulum was exposed and reamed, and a porous acetabular cup was implanted with 15° anteversion and 45° abduction. After screw fixation, a polyethylene liner was inserted. The femoral prosthesis was positioned at 15° anteversion. After testing limb length, an appropriate prosthesis was implanted, followed by assessment of hip flexion and extension range of motion.

Stage 2. PSO from T10 to L4: The patient was placed in the prone position with the abdomen suspended and both knees slightly flexed. After layer-by-layer dissection, polyaxial pedicle screws were implanted at T10–L4 under C-arm guidance. Spinal canal decompression was performed from T12–L2, and bilateral transpedicular osteotomy was performed at L1. Correction was performed until the osteotomy sites of the vertebral body and bilateral laminae were completely closed. After satisfactory fluoroscopy, all nuts were locked, and the dura mater was repaired. Intraoperative neuromonitoring was performed throughout the operation, and no abnormal changes were found in somatosensory and motor evoked potentials.

Stage 3. Contralateral THA was performed, and the procedure was basically identical to that in Stage 1.

The interval between each operation was 2 weeks, mainly due to the following reasons:

1. A single operation was associated with significant blood loss, and the patient's general condition usually recovered well after approximately 2 weeks, with indicators such as albumin and hemoglobin basically returning to the preoperative level;
2. The suture removal time for orthopedic surgeries is usually 2 weeks postoperatively, which does not affect wound healing.

## Postoperative management and follow-Up

Prophylactic antibiotics were administered within 24 h after each operation. The hip drain was removed within 48 h postoperatively, and the spinal drain was removed when the drainage volume was less than 50 ml/24 h. For the hip joint, rehabilitation training was initiated immediately after surgery, including ankle pump exercises and isometric contraction of the quadriceps femoris, 300 times each per day. Within 1 week postoperatively, a soft pillow was placed under the popliteal fossa of the affected limb to maintain hip and knee flexion, thereby reducing nerve tension. After 1 week, the hip and knee flexion angle of the affected limb was gradually reduced. Once the affected limb was fully extended, the patient ambulated with crutches. For the spine, a brace was worn for 3 months after surgery, during which weight-bearing and strenuous activities were prohibited.

**Figure 2:**
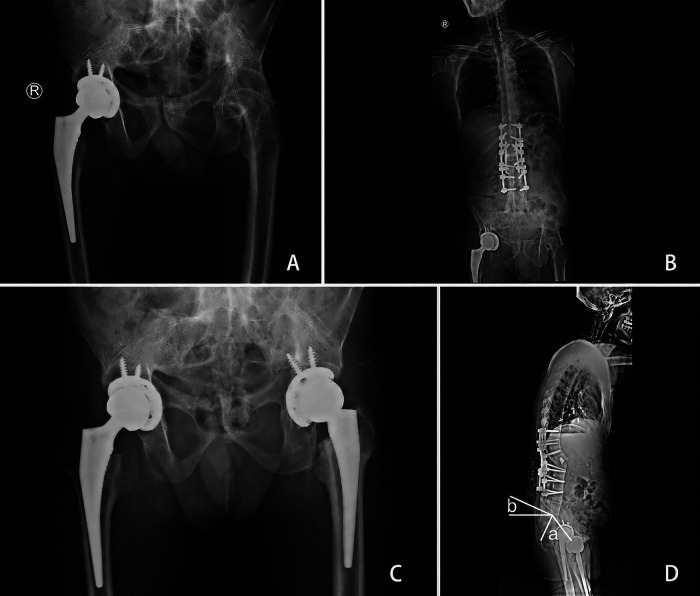
Postoperative x-rays: **(A)** first surgery, right THA, **(B)** significant improvement in scoliosis, **(C)** after the second THA procedure, both hip prostheses are in good position, **(D)** kyphosis has been effectively improved, PI a = 55°, SS b = 21°.

Postoperative re-examination showed that spinal kyphosis and scoliosis were significantly improved, and the patient could resume an upright position. Spinopelvic parameters were as follows: PI = 55°, sacral slope (SS) = 21°, indicating significant correction of pelvic retroversion. The positions of the bilateral hip prostheses were satisfactory (Figure 2). Postoperative efficacy follow-up was mainly performed at 3 months after the final operation, and no complications such as infection, prosthesis displacement, spinal instability, or nerve injury were observed. The bilateral HHS scores increased to 88 points and each hip joint flexion ROM can reach 90°, the ASQoL score decreased to 5 points, and the bilateral hip VAS scores remained 3 points. The patient's bilateral hip joint function was significantly improved; he could walk upright and independently complete daily activities, but there was no significant change in VAS compared with the preoperative period.

## Discussion

Kyphotic deformity of the spine and bony ankylosis of the hip joints are hallmark manifestations of end-stage AS. PSO and THA represent effective surgical interventions for correcting spinal-pelvic malalignment and restoring hip function in these patients. The sequence of THA and PSO is a hot topic in orthopedic discussions. At present, the mainstream view is that prioritizing spinal kyphosis correction can restore the normal physiological position of the pelvis, provide a reliable anatomical reference for the accurate placement of the acetabular prosthesis angle during subsequent hip arthroplasty, and effectively reduce the risk of complications such as hip prosthesis dislocation and loosening. However, for patients with extremely severe spinal kyphosis, anesthesia and intraoperative positioning are extremely difficult (8, 10). Performing hip arthroplasty when the pelvis still has severe tilt and retroversion makes it difficult to accurately grasp the angle of the acetabular prosthesis. Nevertheless, some researchers believe that in certain cases, choosing hip surgery first can effectively promote overall sagittal plane reconstruction, make pelvic parameters more stable, and the advantages in intraoperative blood loss and operation time can improve surgical tolerance and feasibility (9, 11). This case presented several significant technical challenges: 1) Bilateral hip bony fusion accompanied by right femoral flexion and internal rotation, knee flexion, and right spinal curvature made it difficult to place the patient in a reliable and effective prone position and right lateral decubitus position during surgery; 2) The absence of identifiable osseous landmarks due to ankylosis impeded accurate femoral neck resection, acetabular exposure, and precise determination of the hip rotation center; 3) Disruption of the normal spinopelvic relationship—characterized by excessive pelvic retroversion, muscular atrophy, and soft tissue contractures—increased the complexity of acetabular component placement, predisposing to postoperative complications such as dislocation and implant impingement.

From a biomechanical perspective, PI of 55° suggested a preserved pelvic morphology, whereas the postoperative SS of 21° and restoration of upright posture indicated that part of the preoperative retroversion was compensatory rather than structural. This distinction matters because AS patients often have markedly restricted spinopelvic mobility; Oommen et al. found that the sitting-to-standing SS excursion remained limited after THA and that many patients demonstrated “stuck sitting” or “stuck standing” patterns, meaning acetabular orientation must be judged against a narrow functional window instead of a single static radiograph (12). A 2022 systematic review and network meta-analysis showed higher dislocation and other mechanical complication rates in stiff-spine patients after THA, underscoring why definitive cup positioning should be matched to the final spinopelvic alignment whenever feasible (13). These parameters were therefore not only descriptive, but directly influenced our surgical sequencing and acetabular planning strategy.

The right hip was selected first because it was the dominant positional blocker. Its more severe flexion-internal rotation contracture and associated asymmetric lower-limb alignment most directly impaired achievement of a stable left lateral decubitus position for THA and a reproducible prone position for PSO. Releasing this side first was therefore expected to yield the greatest immediate gain in limb extension, decrease the need for forceful positioning, and improve anesthetic and surgical safety before spinal correction. At the same time, retaining the contralateral fused hip temporarily provided a counter-support during osteotomy correction rather than exposing a newly implanted bilateral construct to large corrective moments.

After completion of spinal correction, the spinopelvic sagittal parameters underwent permanent changes. At this point, the contralateral THA was accurately planned and performed based on the final, fixed spinopelvic sagittal parameters. The anteversion and abduction angles of the acetabular cup could be individually set according to the actual postoperative pelvic anteversion/retroversion status to ensure it was in a dynamic “safe zone”, maximizing the reduction of impingement and dislocation risks, and achieving biomechanical matching with the completed spinal correction and the first-stage THA.

Recent outcome data further support this staged matching concept. Functional cup positioning that accounts for pelvic malrotation has demonstrated excellent dislocation-free survival in AS cohorts, suggesting that the last arthroplasty benefits from being planned after the corrected spine-pelvis relationship becomes fixed (14). Similarly, contemporary PSO studies emphasize that preoperative parameters such as PI and global kyphosis directly influence osteotomy planning, reinforcing that hip and spine procedures should be sequenced according to measurable deformity features instead of a rigid one-size-fits-all rule (15).

## Conclusion

For complex AS cases with bilateral hip bony fusion accompanied by femoral flexion, combined with spinal kyphosis and scoliosis (presenting an overall C-shaped curvature in the coronal plane), the limitations of the traditional “hip-first” or “spine-first” strategies can be broken through, and the surgical sequence can be designed according to the individual characteristics of the patient. The “sandwich” staged surgical strategy demonstrated in this case skillfully solved the three major problems of intraoperative positioning, overall alignment reconstruction, and joint parameter adaptation through an ingenious sequence arrangement. This study holds that this strategy follows the biomechanical logic of “unlocking-balance-matching”, effectively reducing surgical risks (especially the risk of postoperative hip dislocation), and ultimately achieving coordinated optimization of the spine-pelvis-hip joint. This experience will provide a new and valuable clinical idea for the management of similar extremely complex hip-spine syndrome cases.

Therefore, the true innovation of the “sandwich” strategy is not merely the order of three operations, but the purpose-specific sequencing of unlocking, rebalancing, and matching. This framework may be particularly valuable in rare AS patients who present not only with sagittal deformity and fused hips, but also with coronal imbalance, because no traditional “hip-first” or “spine-first” pathway alone can simultaneously solve positioning, global alignment, and final cup-orientation problems.

Several limitations warrant more explicit acknowledgment. First, the follow-up was short, so late complications such as wear, delayed dislocation, rod failure, or loosening cannot yet be excluded. Second, because severe fixed deformity precluded complete preoperative standing lateral assessment, we could not quantify all dynamic parameters such as pelvic tilt, sagittal vertical axis, or sitting-standing spinopelvic motion with the same precision used postoperatively. Third, the proposed algorithm is derived from a single asymmetric case and should therefore be interpreted as a hypothesis-generating strategy for selected patients rather than as a universal sequence for every AS-related hip-spine deformity.

## Measurement method

1. Scoliosis angle: Use Cobb method to measure the angle between the upper endplate of C7 vertebra and the lower endplate of L5 vertebra.
2. Kyphosis angle: Thoracolumbar T10-L2, Cobb method is used to measure the angle from the upper endplate of the T10 vertebral body on the head side to the lower endplate of the T2 vertebral body on the tail side.
3. PI：Angle between the perpendicular to the sacral endplate at its midpoint and the line connecting this point to the center of the femoral heads
4. SS: The angle between the tangent line of the sacral endplate and the horizontal line.

Due to the severe pelvic tilt of the patient, it was not possible to obtain a standing lateral pelvic spine image before surgery. Therefore, we only obtained the PI angle from the patient's sitting image.

## Data Availability

The original contributions presented in the study are included in the article/Supplementary Material, further inquiries can be directed to the corresponding author/s.
